# Yoga for psychological outcomes and quality of life in non-cystic fibrosis bronchiectasis: a controlled study

**DOI:** 10.3389/fpsyt.2025.1695692

**Published:** 2025-10-23

**Authors:** Lele Hei, Yue Zhang, Yanyan Si, Lili Bai

**Affiliations:** ^1^ General Medicine Department, Yan’an People’s Hospital, Yan’an, China; ^2^ Third Ward, Department of Acupuncture and Moxibustion, Shaanxi Hospital of Traditional Chinese Medicine, Xi’an, China; ^3^ Department of Critical Care Medicine, Yan ‘an Hospital of Chinese Medicine, Yan’an, China; ^4^ Second Ward, Department of Respiratory and Critical Care Medicine, Yan ‘an People’s Hospital, Yan’an, China

**Keywords:** non-cystic fibrosis bronchiectasis, yoga intervention, quality of life, mental health, stress, anxiety, depression

## Abstract

**Objectives:**

This study explored the potential impact of a structured yoga program over an 8-week period on aspects of psychological health and health-related quality of life (HRQoL) among individuals diagnosed with non-cystic fibrosis bronchiectasis.

**Methods:**

A prospective, controlled clinical study was carried out from January 2023 to January 2024. Sixty-nine adults with clinically stable non-cystic fibrosis bronchiectasis were recruited and assigned randomly to either a yoga intervention group (n = 24) or a control group receiving standard care (n = 45). The yoga group engaged in supervised online sessions three times weekly, while the control group continued with routine clinical management without any added exercise regimen. HRQoL was measured using the Quality of Life-Bronchiectasis (QOL-B) questionnaire. Psychological variables were assessed through the Short Form-36 (SF-36), Hospital Anxiety and Depression Scale (HADS), and the Perceived Stress Scale-10 (PSS-10). Evaluations took place at baseline and after the 8-week period. Statistical analyses were applied based on data distribution, with significance determined at a threshold of *P* < 0.05.

**Results:**

Participants undergoing yoga demonstrated notable improvements in select QOL-B domains, particularly in areas such as respiratory symptoms, emotional aspects, and vitality, when compared to the control group (*P* < 0.05). SF-36 results suggested gains in both physical and mental summary scores in the yoga group; however, the control group showed no comparable changes. Psychological assessments indicated reduced anxiety and depression scores, as well as lower perceived stress levels, in those practicing yoga (*P* < 0.01). Group comparisons suggested that the yoga intervention was more favorable than standard care in influencing the measured outcomes. However, the study was limited by its relatively small sample size, short intervention duration, and reliance on self-reported data, which may affect the generalizability and long-term sustainability of the observed benefits.

**Conclusion:**

An 8-week structured yoga program may contribute to improvements in psychological well-being and quality of life among patients with non-cystic fibrosis bronchiectasis. Yoga might represent a complementary approach alongside conventional management, though its role requires additional exploration.

## 1 Introduction

Bronchiectasis is a long-standing respiratory condition that tends to progress over time and is typically marked by abnormal widening of the bronchial airways. This structural alteration is often associated with ongoing airway inflammation and disruptions in normal mucociliary function. As a result, individuals may experience symptoms such as a persistent cough, frequent sputum production, breathlessness, and general fatigue, along with a tendency toward repeated lower respiratory tract infections (1–3). Once considered an orphan disease, non-cystic fibrosis bronchiectasis has experienced a resurgence in clinical relevance, with epidemiological studies reporting increasing prevalence and incidence across diverse populations, particularly in older adults and those with coexisting pulmonary or systemic diseases such as chronic obstructive pulmonary disease (COPD), asthma, or rheumatoid arthritis (4, 5).

Beyond the physical manifestations, bronchiectasis imposes a considerable psychological and emotional burden on patients. In individuals with bronchiectasis, factors such as ongoing respiratory symptoms, occasional exacerbation episodes, extended courses of antibiotic treatment, and the routine requirement for airway clearance techniques may be associated with reductions in health-related quality of life (HRQoL) and increased levels of psychological burden over time (6–8). Symptoms of anxiety and depression are relatively frequent among individuals with bronchiectasis, though they may remain unrecognized or insufficiently managed in routine care. These psychological conditions have been linked, to varying degrees, with heightened symptom awareness, limitations in physical functioning, less favorable clinical trajectories, and a potential increase in healthcare resource use (9, 10). Furthermore, perceived stress is an emerging dimension in chronic respiratory disease management, linked to autonomic dysregulation and systemic inflammation, which may further exacerbate the disease trajectory (11).

Although pharmacological treatment, airway clearance techniques, pulmonary rehabilitation, and infection control form the cornerstone of bronchiectasis management, these approaches predominantly target physiological parameters (12) and do not fully address the emotional or psychosocial dimensions of disease burden. Within the broader context of managing chronic respiratory conditions, there is a growing, though still evolving, interest in incorporating complementary and holistic approaches. These strategies, which may support self-regulatory capacity, emotional adaptation, and aspects of quality of life, are increasingly being explored as potential adjuncts to conventional care, rather than as central elements of treatment (13, 14).

Yoga is a mind-body practice that combines physical movements (asanas), breathing exercises (pranayama), and meditative or relaxation techniques (e.g., dhyana and dharana). While structured in nature, its components and applications may vary depending on the context and setting (15, 16). Over the past several years, yoga has been noted with growing interest within integrative medicine, primarily due to its relative affordability, general accessibility, and its potential to influence both physical and psychological parameters. However, its role remains subject to ongoing investigation, particularly regarding its applicability across diverse clinical populations (17–19). In the context of chronic respiratory diseases like asthma and COPD, yoga has been explored as a complementary approach. Some studies suggest it may contribute to modest improvements in parameters such as pulmonary function, symptom perception (including breathlessness and fatigue), and psychological well-being (20, 21). However, these findings are not universally consistent, and the variability in results may stem from differences in intervention protocols, study designs, patient populations, and limitations such as small sample sizes and randomization issues in previous studies (22–24). It has been reported (22) that both yoga therapy (T-YT) and pulmonary rehabilitation (T-PR) significantly improved exercise capacity, symptom scores, quality of life, and depression and anxiety in COPD patients, with no significant differences between the two groups, suggesting that outcome variability may stem from differences in intervention design. Similarly, a modified Iyengar yoga program benefited some COPD patients, with greater improvements in the yoga group compared to the wait-list control (24). However, responses varied, highlighting the need for further research to identify predictors of success (24). Additionally, potential influences on autonomic regulation and aspects of quality of life have been reported, although findings remain heterogeneous and are influenced by variability in intervention protocols and patient populations (23, 25). The proposed mechanisms, including improvement in ventilatory mechanics, increased respiratory muscle strength, attenuation of sympathetic overactivity, reduction in systemic inflammatory markers, and enhancement of mind–body awareness, have been suggested, but these mechanisms have not been uniformly validated across studies (26, 27).

Despite accumulating evidence supporting yoga’s benefits in chronic respiratory diseases, its therapeutic role in non-cystic fibrosis bronchiectasis remains underexplored. Robust clinical trials specifically examining the role of yoga as an adjunctive intervention in individuals with non-cystic fibrosis bronchiectasis remain limited. In particular, evidence pertaining to its potential influence on psychological well-being and quality of life measures tailored to this condition is relatively sparse, despite these domains often being negatively impacted in this population. Given the chronic disease course, high psychological burden, and the demand for holistic, sustainable care beyond pharmacotherapy, yoga may serve as a valuable adjunct in multidisciplinary management. However, further research is necessary to fully elucidate its effectiveness and clarify its mechanisms in this context. Accordingly, this study sought to explore whether an 8-week structured yoga program might be associated with changes in psychological parameters and aspects of health-related quality of life among individuals with clinically stable bronchiectasis.

## 2 Methods

### 2.1 Study design

This research was carried out in the Department of Pulmonary Medicine at Yanbian People’s Hospital. Individuals with a confirmed diagnosis of non-cystic fibrosis bronchiectasis who visited the outpatient service between January 2023 and January 2024 were considered for potential inclusion. The study followed a prospective, controlled design and received approval from the Institutional Ethics Committee of Yanan People’s Hospital. All study-related procedures adhered to the principles outlined in the Declaration of Helsinki. Prior to participation, written informed consent was obtained from each individual.

### 2.2 Participants enrollment

All participants were diagnosed with non-cystic fibrosis bronchiectasis and underwent clinical and radiological assessment prior to enrollment. Eligible participants met the following conditions: (1) Age greater than 18 years. (2) Diagnosed with non-cystic fibrosis bronchiectasis for at least 3 months. (3) No regular exercise participation in the past 3 months. (4) No other chronic respiratory diseases. (5) Clinically stable with no current exacerbation. (6) No physical or cognitive impairments affecting study participation. (7) Able to understand instructions and questionnaires. (8) Provided written informed consent. Exclusion criteria included: (1) Acute exacerbation or antibiotic use within 4 weeks before the study; (2) Presence of other chronic pulmonary or unstable systemic diseases. (3) Inability to comply with the study protocol for any reason.

### 2.3 Randomization

**Figure 1:**
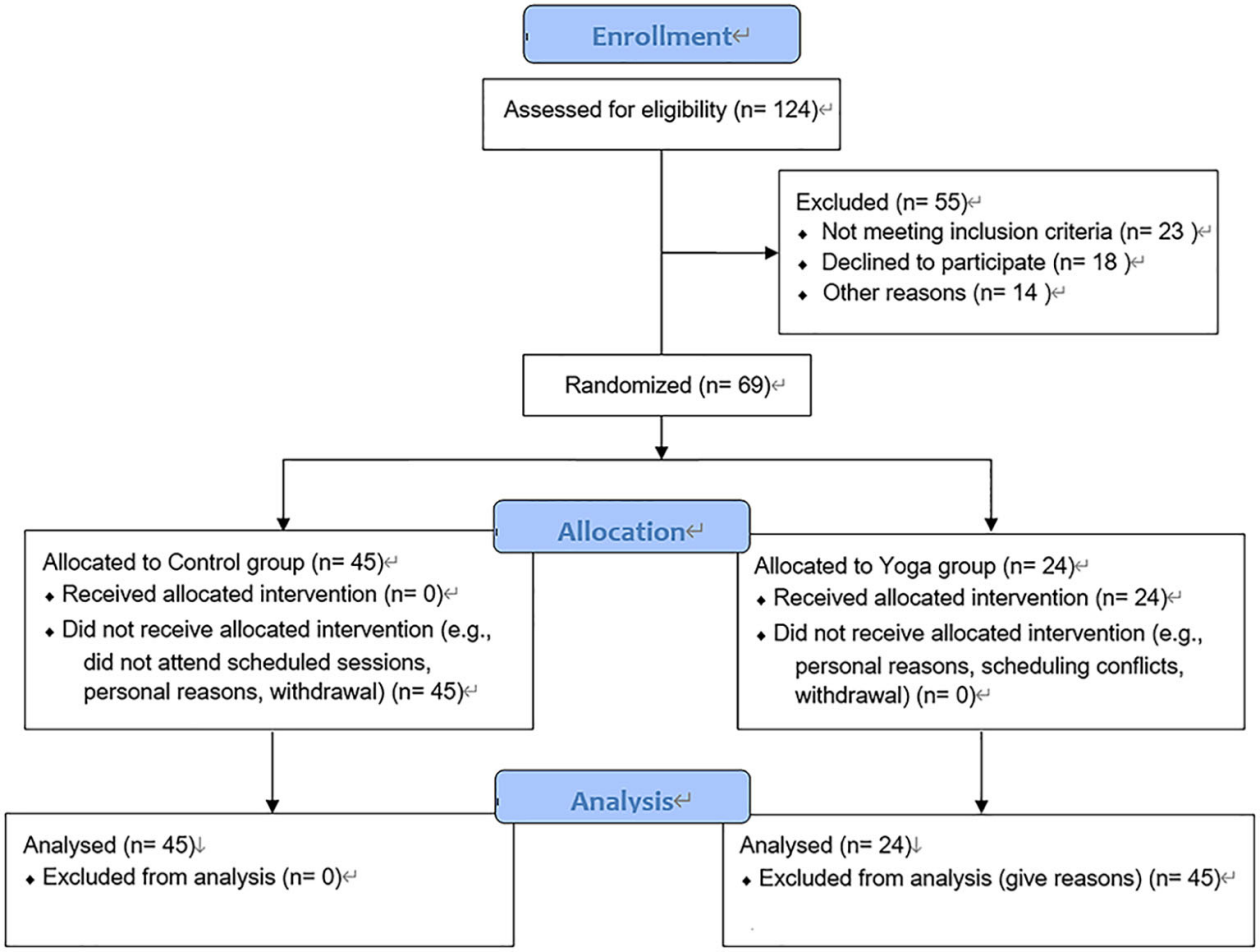
CONSORT diagram and detail allocation concealment, withdrawals/dropouts, and reasons for group size imbalance.

Between January 2023 and January 2024, a total of 69 individuals with a confirmed diagnosis of non-cystic fibrosis bronchiectasis who met the study’s inclusion criteria and provided informed consent were recruited. Participants were randomly allocated to either a yoga group (n = 24) or a control group (n = 45). The control group continued receiving routine clinical care without any additional structured physical activity, while the yoga group participated in a guided yoga program under supervision. Randomization was performed using an online tool (Research Randomizer: https://www.randomizer.org/), and group assignments were concealed in sealed, opaque envelopes. Each participant selected an envelope, with allocation revealed by a member of the research team not involved in outcome evaluation, ensuring allocation concealment. While blinding of participants was not feasible due to the nature of the intervention, outcome assessors were blinded to group assignments to minimize bias. Final group composition was determined by participants’ adherence to the intervention following random allocation (**Figure 1**).

### 2.4 Intervention procedures

The yoga component was implemented over an 8-week period, during which participants were invited to join supervised online sessions approximately three times per week, each lasting around 65 minutes. A trained researcher led the sessions and collected contact information to ensure continuity. Participants were grouped (6–8 per group) based on availability, and instructional materials, videos, and printed booklets prepared in line with existing literature were distributed (28). The structure of each session generally followed commonly used yoga practices, incorporating elements such as breathing exercises (pranayama), physical postures (asanas), and relaxation or meditative components (29, 30). Each session comprised the following: (1) Around 10 minutes of standing breath-based practices: For example, Tadasana Pranayama (standing mountain pose with controlled breathing) and Ardha Kati Chakrasana (half spinal twist), repeated up to three times. These practices aimed to improve posture and breath control while enhancing focus and relaxation. (2) Approximately 10 minutes of seated breathing exercises: Involving postures such as Shashankasana Pranayama (moon pose), Vakrasana (twisted posture), and Ardha Matsyendrasana (half spinal twist). These exercises were intended to open the chest, improve lung capacity, and promote relaxation. Each posture was held for about 3–5 breaths and repeated up to three times. (3) 10 minutes dedicated to asana-integrated breathing (Asana Pranayama): This segment combined physical postures and controlled breathing to improve flexibility, lung function, and mental clarity. For example, participants would engage in poses like Bhujangasana (cobra pose) or Setu Bandhasana (bridge pose), synchronized with slow, deep breathing. (4) A final 15-minute segment emphasizing extended seated breathing: During this segment, earlier sequences were revisited as appropriate, and extended breath retention was incorporated. Breath awareness was emphasized to reduce stress and promote mindfulness. (5) 10 minutes of alternate nostril and forceful breathing: Techniques such as Bhastrika (bellows breath), Nadishodhana (alternate nostril breathing), and Suryanuloma Viloma (breathing with nostril alternation) were practiced for 5 rounds (**Table 1**). These breathing techniques were designed to enhance lung function, improve oxygenation, and reduce stress. (6) 10 minutes of deep relaxation: This final component included practices like Dharana (focused concentration), Dhyana (meditative awareness), and Samadhi (complete absorption) to relax the body and mind, leading to a meditative state that supported emotional balance and mental clarity.

**Table 1:** Intervention table of yoga.

Sr. no.	Name of independent variable (yoga practice)	Time (minutes)	Rest time (minutes)	Total rounds	Total time (minutes)
1	Standing Breath-based Practices (e.g., Tadasana Pranayama, Ardha Kati Chakrasana)	8	2	3	10
2	Seated Breathing Exercises (e.g., Shashankasana Pranayama, Vakrasana)	8	2	3	10
3	Asana-integrated Breathing (e.g., Bhujangasana, Setu Bandhasana)	8	2	3	10
4	Extended Seated Breathing (Breath retention, mindfulness)	12	3	N/A	15
5	Alternate Nostril and Forceful Breathing (e.g., Bhastrika, Nadishodhana, Suryanuloma Viloma)	8	2	5	10
6	Deep Relaxation (e.g., Dharana, Dhyana, Samadhi)	8	2	N/A	10
Total	Full Yoga Session	60	10	N/A	70

N/A, Not Applicable.

To ensure standardization across sessions, all sessions were led by the same trained researcher, who followed a strict protocol to minimize variability. Additionally, instructional materials were standardized and provided consistently across all sessions. Participants were encouraged to provide feedback, and all remained under pulmonologist supervision. Mucoactive agents were withheld unless clinically indicated. Participants in the control group did not receive any additional structured intervention during the study period. However, all individuals, regardless of group allocation, were offered standard educational resources and general guidance regarding the importance of respiratory physiotherapy as part of routine bronchiectasis management. Adherence was closely monitored throughout the intervention. Notably, there were no dropouts from the yoga group. All participants attended the scheduled sessions as per the protocol. This highlights the feasibility and participant engagement with the intervention.

### 2.5 Clinical data collection

Baseline clinical data were collected prior to group allocation using standardized forms by trained staff. Collected variables included demographic information (age, sex, BMI, smoking status, and education level) and medical history (diabetes, hypertension, asthma, rhinosinusitis, depression, obstructive sleep apnea, and gastroesophageal reflux). Respiratory symptoms such as dyspnea, wheezing, cough, phlegm, and chest tightness (atresia) were recorded through patient report and clinical evaluation. Bronchiectasis severity was assessed using: (1) The FACED score was used as a multidimensional index to describe disease characteristics, taking into account patient age, percent-predicted FEV_1_, colonization with Pseudomonas aeruginosa, the number of lobes involved as seen on high-resolution CT (HRCT), and level of dyspnea based on the modified Medical Research Council (mMRC) scale. Based on the composite score, patients were generally categorized into three groups—mild (0–2), moderate (3–4), or more severe disease (5–7)—primarily for descriptive purposes (31). (2) Bronchiectasis Severity Index (BSI), which includes age, BMI, FEV_1_, exacerbation history, prior hospitalization, dyspnea, microbiology, radiological severity, and hemoptysis. The BSI was used to provide an overall indication of disease burden, with score ranges commonly interpreted as reflecting mild (0–4), moderate (5–8), or more severe clinical presentations (≥9) (32). Pulmonary function data were obtained via standardized spirometry. All data were anonymized and securely stored.

### 2.6 Outcome assessment

This study primarily aimed to explore changes in HRQoL and psychological well-being over the course of the 8-week observation period. These parameters were assessed at baseline and following the intervention using a set of validated, self-reported questionnaires. To minimize potential bias, assessments were carried out under the oversight of trained research personnel who remained unaware of participants’ group assignments.

#### 2.6.1 Quality of life assessment

HRQoL was evaluated using the Quality of Life–Bronchiectasis (QOL-B) questionnaire, a condition-specific instrument developed to reflect the lived experiences of individuals with bronchiectasis (33). The QOL-B encompasses several domains, such as respiratory symptoms, physical and emotional functioning, vitality, role limitations, social engagement, general health perceptions, and perceived treatment burden. Each domain yields a score from 0 to 100, where higher values generally correspond to more favorable outcomes. For this study, a Chinese-translated version of the QOL-B—previously examined in bronchiectasis cohorts—was employed.

#### 2.6.2 Psychological outcomes assessment

Psychological well-being was evaluated using three validated instruments:

1. Short Form-36 Health Survey (SF-36) (34): to provide a broad overview of perceived health status, the physical component summary (PCS) and mental component summary (MCS) scores were considered. These composite scores reflect general physical well-being and psychological outlook, respectively. Each domain generates a value between 0 and 100, where higher scores are typically interpreted as indicative of more favorable self-reported health.
2. Hospital Anxiety and Depression Scale (HADS) (35): This 14-item instrument includes two subscales, one for anxiety and one for depression, each comprising 7 items, with scores on each subscale ranging from 0 to 21. Higher values may be interpreted as suggestive of greater symptom severity. In addition, a combined HADS score was derived to provide a general indication of emotional burden, although its use in capturing overall psychological distress should be considered with contextual caution.
3. Perceived Stress Scale-10 (PSS-10) (36): This instrument is designed to gauge self-reported stress levels experienced over the preceding month, incorporating a mix of positively and negatively framed items. The total score ranges from 0 to 40, with higher values generally associated with a greater sense of perceived stress, though interpretation may vary depending on individual context and baseline characteristics.

Questionnaires were administered at two assessment points: prior to randomization (baseline) and following completion of the 8-week intervention period. Participants were encouraged to complete the forms independently, with assistance available if clarification was needed. To reduce potential bias, data collection was conducted by staff members who were unaware of participants’ group allocation. The measurement tools employed had previously demonstrated acceptable levels of reliability and internal consistency within Chinese populations.

### 2.7 Statistical analysis

All statistical procedures were carried out using IBM SPSS Statistics for Windows, version 26.0 (IBM Corp., Armonk, NY, USA). Descriptive analyses were applied to summarize baseline characteristics and outcome measures. Continuous variables were reported as means with standard deviations (SD) when normally distributed, or as medians with interquartile ranges (IQR) when distributional assumptions were not met. Categorical data were presented as frequencies and percentages. Normality was assessed using the Shapiro–Wilk test. For comparisons between groups, independent samples t-tests were employed for continuous variables with approximately normal distributions, while the Mann–Whitney U test was used when data deviated from normality. Categorical variables were analyzed using the Chi-square test or Fisher’s exact test, depending on distribution and expected cell counts. Changes within groups from pre- to post-intervention were examined using paired samples t-tests for normally distributed data and the Wilcoxon signed-rank test for non-parametric comparisons. A two-tailed significance threshold of *P* < 0.05 was applied for all tests. To control for Type I error from multiple comparisons, Bonferroni correction was applied by adjusting the significance threshold (0.05) based on the total number of comparisons. For QOL-B, with 16 comparisons (8 variables × 2 tests), the threshold was set to 0.003 (0.05 ÷ 16). For **Table 2** (SF-36, HADS, PSS-10), with 18 comparisons (9 variables × 2 tests), the threshold was set to 0.003 (0.05 ÷ 18). Statistical significance was determined using the adjusted p-values, with results considered significant if the p-value was below the respective threshold.

**Table 2:** Psychological outcomes of control and yoga groups assessed by SF-36, HADS and PSS-10.

Indices	Control group (*n* = 45)	Yoga group (*n* = 24)	*P* ^a^
SF-36			
Physical (Pre)	53.00 (47.50, 58.00)	49.00 (44.25, 57.00)	0.13
Physical (Post)	52.00 (47.00, 57.00)	57.50 (49.75, 66.50)	0.013
*P* ^b^	0.574	0.006	
Mental (Pre)	54.00 (48.50, 60.50)	53.50 (49.00, 59.00)	0.791
Mental (Post)	52.00 (47.50, 62.00)	62.00 (57.25, 68.50)	<0.001
*P* ^b^	0.608	<0.001	
Total score (Pre)	55.00 (48.00, 58.50)	54.00 (49.25, 58.75)	0.738
Total score (Post)	57.00 (49.50, 62.00)	66.50 (59.25, 74.75)	<0.001
*P* ^b^	0.07	<0.001	
HADS			
Anxiety (Pre)	6.00 (5.00, 7.00)	6.00 (5.00, 7.00)	0.28
Anxiety (Post)	6.00 (4.00, 7.00)	4.00 (2.00, 6.00)	0.029
*P* ^b^	0.389	0.037	
Depression (Pre)	5.00 (3.00, 7.00)	6.00 (4.00, 8.75)	0.228
Depression (Post)	6.00 (4.50, 7.00)	4.50 (2.00, 6.00)	0.006
*P* ^b^	0.15	0.012	
Total score (Pre)	9.00 (7.00, 11.00)	10.00 (8.25, 14.00)	0.09
Total score (Post)	9.00 (8.00, 11.00)	7.50 (4.25, 9.00)	0.002
*P* ^b^	0.717	<0.001	
PSS-10			
Positive (Pre)	6.00 (4.00, 8.00)	5.00 (3.00, 7.75)	0.301
Positive (Post)	6.00 (5.00, 7.50)	3.00 (2.00, 5.00)	<0.001
*P* ^b^	1	0.015	
Negative (Pre)	4.00 (2.00, 6.50)	5.00 (4.00, 7.00)	0.097
Negative (Post)	5.00 (3.00, 6.00)	3.50 (2.00, 5.00)	0.013
*P* ^b^	0.613	0.003	
Total score (Pre)	8.00 (6.00, 11.00)	9.00 (6.25, 10.00)	0.658
Total score (Post)	8.00 (6.00, 9.50)	5.50 (3.00, 7.00)	<0.001
*P* ^b^	0.425	<0.001	

SF-36, Short Form-36 Health Survey; HADS, Hospital Anxiety and Depression Scale; PSS-10, Perceived Stress Scale-10.

Pre: pre-intervention assessment; Post: post-intervention assessment.

*P* ^a^: between-group comparison (independent samples t-test); *P* ^b^: within-group comparison (paired samples t-test).

*P* < 0.05 considered statistically significant.

Group comparability at baseline was reviewed prior to further analysis. Primary outcomes were exploratory in nature and focused on changes in quality of life, as assessed by the QOL-B questionnaire, along with shifts in psychological parameters measured using the SF-36, HADS, and PSS-10. The intervention’s potential influence was evaluated by examining both within- and between-group changes over the 8-week period.

## 3 Results

### 3.1 Baseline characteristics comparison

A total of 69 individuals diagnosed with bronchiectasis were enrolled in the study and subsequently assigned to either a control group (n = 45) or a yoga group (n = 24) as part of the intervention framework. Baseline demographics, including age (44.24 ± 4.85 vs. 45.21 ± 4.73 years), BMI (25.91 ± 2.28 vs. 26.73 ± 1.26 kg/m²), and gender distribution (male: 53.33% vs. 45.83%), were comparable between groups (all P > 0.05). Smoking status also showed no significant difference, with similar proportions of current (46.67% vs. 62.50%) and former smokers (53.33% vs. 37.50%) across the control and yoga groups (P = 0.312). Education level was similarly distributed (P = 0.476), with the majority of patients in both groups having attained university-level education (62.22% vs. 62.50%). The prevalence of comorbid conditions, such as diabetes (22.22% in the control group vs. 16.67% in the yoga group), hypertension (48.89% vs. 37.50%), asthma (17.78% vs. 29.17%), rhinosinusitis (24.44% vs. 37.50%), depression (4.44% vs. 16.67%), obstructive sleep apnea (8.89% vs. 20.83%), and gastroesophageal reflux (2.22% vs. 8.33%), varied across participants but did not show statistically significant differences between groups (all P > 0.05). Clinical severity profiles, based on assessments using the FACED score and BSI, appeared generally comparable between groups, with no statistically significant differences observed. Mild FACED scores (0–2 points) were observed in 57.78% of control and 62.50% of yoga participants, while moderate scores (3–4 points) were present in 42.22% and 37.50%, respectively (P = 0.799). Based on BSI classification, the proportions of participants categorized as having mild (20.00% in the control group vs. 16.67% in the yoga group), moderate (46.67% vs. 41.67%), or severe disease (33.33% vs. 41.67%) were broadly distributed across both groups, with no statistically meaningful differences (P = 0.797). Similarly, the prevalence of common presenting symptoms—such as dyspnea (86.67% vs. 95.83%), chest tightness or atresia (57.78% vs. 66.67%), wheezing (53.33% vs. 70.83%), cough (64.44% vs. 75.00%), and sputum production (66.67% vs. 70.83%)—did not significantly differ between groups (all P > 0.05). A summary of these baseline characteristics is provided in **Table 3**.

**Table 3:** Baseline characteristics of control and yoga groups.

Indices	Control group (*n* = 45)	Yoga group (*n* = 24)	*P*
Age (Years)	44.24 ± 4.85	45.21 ± 4.73	0.430
BMI (kg/m^2^)	25.91 ± 2.28	26.73 ± 1.26	0.106
Gender [n(%)]			0.618
Male	24 (53.33)	11 (45.83)	
Female	21 (46.67)	13 (54.17)	
Smoking status [n(%)]			0.312
Current	21 (46.67)	15 (62.50)	
Former	24 (53.33)	9 (37.50)	
Education [n(%)]			0.476
Elementary	8 (17.78)	2 (8.33)	
High school	9 (20.00)	7 (29.17)	
University	28 (62.22)	15 (62.50)	
Comorbidities [n(%)]			
Diabetes	10 (22.22)	4 (16.67)	0.756
Hypertension	22 (48.89)	9 (37.50)	0.449
Asthma	8 (17.78)	7 (29.17)	0.36
Rhinosinusitis	11 (24.44)	9 (37.50)	0.278
Depression	2 (4.44)	4 (16.67)	0.173
Obstructive sleep apnea	4 (8.89)	5 (20.83)	0.259
Gastroesophageal reflux	1 (2.22)	2 (8.33)	0.276
FACED score [n(%)]			0.799
Mild (0–2 points)	26 (57.78)	15 (62.50)	
Moderate (3–4 points)	19 (42.22)	9 (37.50)	
BSI index [n(%)]			0.797
Mild	9 (20.00)	4 (16.67)	
Moderate	21 (46.67)	10 (41.67)	
Severe	15 (33.33)	10 (41.67)	
Cause of admission [n(%)]			
Dyspnea	39 (86.67)	23 (95.83)	0.408
Atresia	26 (57.78)	16 (66.67)	0.606
Wheeze	24 (53.33)	17 (70.83)	0.202
Cough	29 (64.44)	18 (75.00)	0.427
Phlegm	30 (66.67)	17 (70.83)	0.792

BMI, Body Mass Index; FACED, FEV1–Age–Chronic colonization–Extension–Dyspnea; BSI, Bronchiectasis Severity Index.

### 3.2 Improvement of Quality of Life outcomes following a yoga program in individuals with bronchiectasis

**Figure 2:**
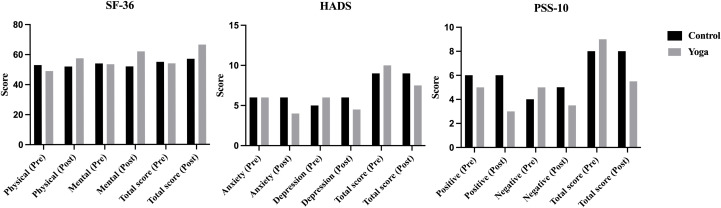
Life quality of control and yoga groups assessed by QOL-B.

Given the comparable baseline characteristics between groups, analyses explored quality of life changes after the intervention (**Table 4**, **Figure 2**). At baseline, QOL-B measures, including respiratory symptoms, physical functioning, vitality, role functioning, health perceptions, emotional and social functioning, and treatment burden, did not differ significantly between groups (all P > 0.05, corrected P > 0.003). After 8 weeks, the yoga group showed improvements in several QOL-B domains compared to controls. Notably, respiratory symptoms in the yoga group increased to [84.50 (79.25, 90.75)] vs. control [72.00 (70.00, 81.00)] (P < 0.001, corrected P < 0.003). Similar trends were observed in physical symptoms [96.50 (88.25, 99.00) vs. 84.00 (77.00, 90.50), P < 0.001, corrected P < 0.003], vitality [88.50 (77.25, 94.00) vs. 63.00 (58.00, 66.50), P < 0.001, corrected P < 0.003], and role functioning [92.00 (88.50, 95.00) vs. 78.00 (72.50, 83.00), P < 0.001, corrected P < 0.003]. Health perceptions were also higher in the yoga group [72.50 (68.00, 78.75)] vs. control [64.00 (55.00, 68.50)] (P < 0.001, corrected P < 0.003). Emotional and social functioning scores were higher in the yoga group: emotional functioning [89.00 (83.25, 94.00) vs. 73.00 (67.00, 78.50)]; social functioning [87.50 (82.25, 91.00) vs. 66.00 (56.50, 74.00)], both P < 0.001, corrected P < 0.003. Additionally, treatment burden was lower in the yoga group [96.50 (87.00, 100.00) vs. 84.00 (78.00, 88.00), P < 0.001, corrected P < 0.003]. Within-group analyses showed improvements in the yoga group across all domains (all P < 0.001, corrected P < 0.003), while the control group showed limited changes, with only a small decline in vitality (P = 0.021, corrected P < 0.003). These outcomes, summarized in **Table 4** and **Figure 2**, suggest potential benefits of yoga in quality of life, but further validation in larger, more diverse samples is recommended.

**Table 4:** Life quality of control and yoga groups assessed by QOL-B.

Indices	Control group (*n* = 45)	Yoga group (*n* = 24)	*P* ^a^
Respiratory symptoms (Pre)	75.00 (67.50, 79.50)	75.50 (66.50, 80.75)	0.845
Respiratory symptoms (Post)	72.00 (70.00, 81.00)	84.50 (79.25, 90.75)	<0.001
*P* ^b^	0.99	<0.001	
Physical symptoms (Pre)	83.00 (76.00, 87.00)	80.00 (76.00, 86.50)	0.541
Physical symptoms (Post)	84.00 (77.00, 90.50)	96.50 (88.25, 99.00)	<0.001
*P* ^b^	0.161	<0.001	
Vitality (Pre)	67.00 (63.00, 72.00)	65.00 (61.00, 74.75)	0.453
Vitality (Post)	63.00 (58.00, 66.50)	88.50 (77.25, 94.00)	<0.001
*P* ^b^	0.021	<0.001	
Role functioning (Pre)	81.00 (74.00, 87.00)	79.50 (72.50, 82.00)	0.211
Role functioning (Post)	78.00 (72.50, 83.00)	92.00 (88.50, 95.00)	<0.001
*P* ^b^	0.085	<0.001	
Health perceptions (Pre)	63.00 (52.50, 69.00)	59.00 (54.25, 66.25)	0.496
Health perceptions (Pre)	64.00 (55.00, 68.50)	72.50 (68.00, 78.75)	<0.001
*P* ^b^	0.579	<0.001	
Emotional functioning (Pre)	69.00 (60.50, 75.00)	69.50 (62.25, 77.00)	0.496
Emotional functioning (Post)	73.00 (67.00, 78.50)	89.00 (83.25, 94.00)	<0.001
*P* ^b^	0.059	<0.001	
Social functioning (Pre)	68.00 (61.50, 75.00)	69.00 (60.75, 73.00)	0.484
Social functioning (Post)	66.00 (56.50, 74.00)	87.50 (82.25, 91.00)	<0.001
*P* ^b^	0.235	<0.001	
Treatment burden (Pre)	86.00 (76.50, 93.50)	91.75 (81.00, 99.50)	0.341
Treatment burden (Post)	84.00 (78.00, 88.00)	96.50 (87.00, 100.00)	<0.001
*P* ^b^	0.481	<0.001	

QOL-B, quality of Life bronchiectasis questionnaire.

0 = worst symptoms; 100 = no symptoms.

Pre: pre-intervention assessment; Post: post-intervention assessment.

*P* ^a^: between-group comparison (independent samples t-test); *P* ^b^: within-group comparison (paired samples t-test).

*P* < 0.05 considered statistically significant.

### 3.3 Improvement of psychological outcomes following a yoga program in individuals with bronchiectasis

**Figure 3:**
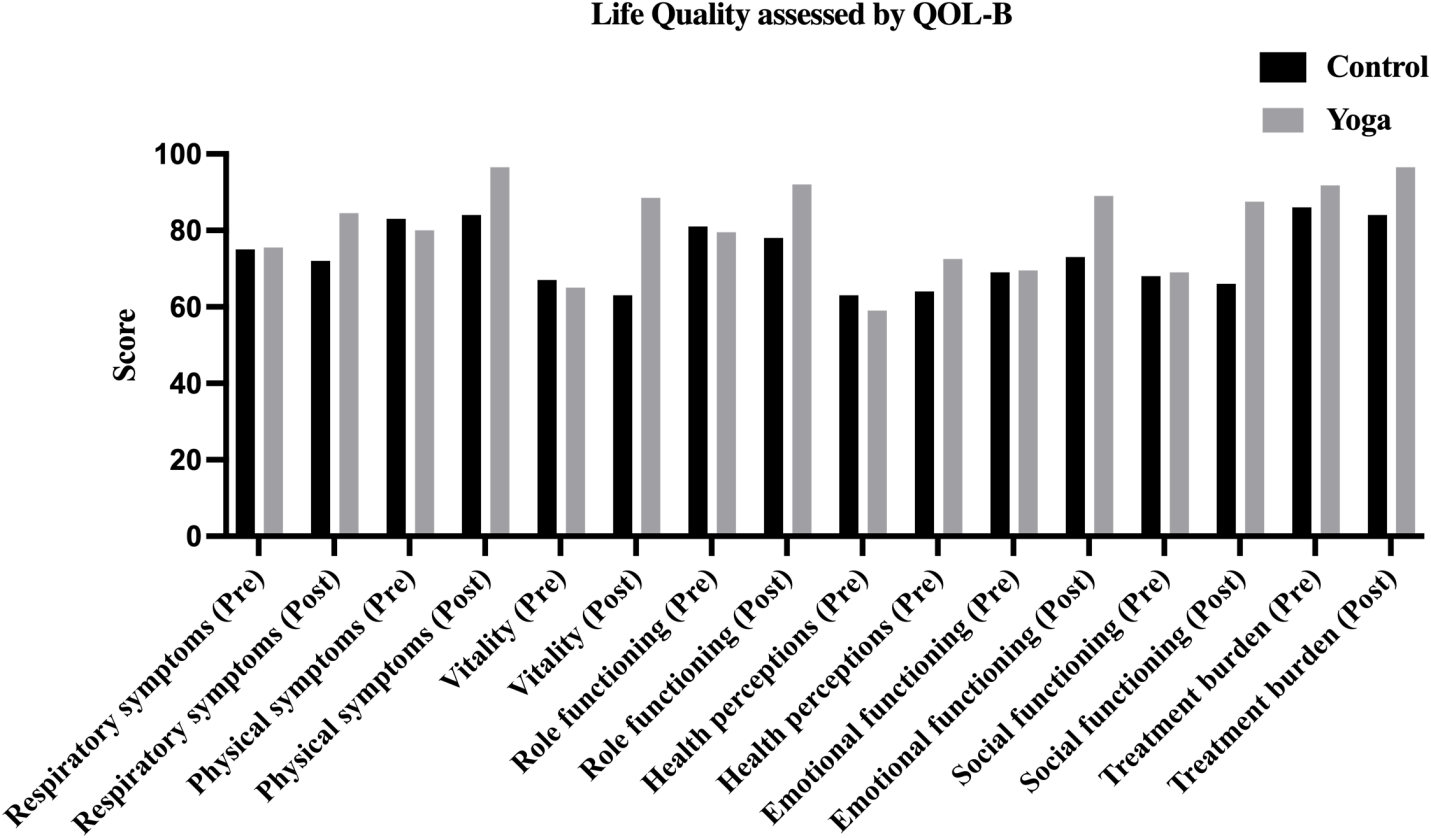
Psychological outcomes of control and yoga groups assessed by SF-36, HADS and PSS-10.

After evaluating quality of life, psychological parameters were assessed using SF-36, HADS, and PSS-10 (**Table 2**, **Figure 3**). At baseline, no significant differences were observed between groups across psychological domains (all P > 0.05, corrected P > 0.003), indicating comparable psychological profiles prior to the intervention. Post-intervention, the yoga group showed improvements in several measures. For SF-36, the yoga group exhibited higher post-intervention scores in the mental health component [62.00 vs. 52.00, P < 0.001, corrected P < 0.003] and the total score [66.50 vs. 57.00, P < 0.001, corrected P < 0.003]. The physical health component showed a trend towards improvement but was not statistically significant after Bonferroni correction [57.50 vs. 52.00, P = 0.013, corrected P > 0.003]. In the HADS, the yoga group had significantly lower total scores [7.50 vs. 9.00, P = 0.002, corrected P < 0.003], with anxiety [P = 0.029, corrected P > 0.003] and depression [P = 0.006, corrected P > 0.003] showing no significant difference. In the PSS-10, the yoga group reported reductions in positive stress items [3.00 vs. 6.00, P < 0.001, corrected P < 0.003] and total stress [5.50 vs. 8.00, P < 0.001, corrected P < 0.003], but no significant difference was found in negative stress items [P = 0.013, corrected P > 0.003]. These findings suggest potential psychological benefits of yoga, though caution is needed due to the study’s size and design. No significant changes were observed in the control group (P > 0.05). Full data are summarized in **Table 2**.

## 4 Discussion

This prospective, controlled study provides preliminary evidence suggesting that an 8-week structured yoga program may be associated with improvements in HRQoL and selected psychological parameters among individuals with stable non-cystic fibrosis bronchiectasis. These observations were made in the context of unchanged pharmacologic and respiratory therapy regimens during the intervention period. While the findings point to the potential utility of yoga as a low-cost, non-pharmacologic complement to standard care, further investigation in larger, more diverse cohorts is warranted to better define its role within multidisciplinary bronchiectasis management.

Among the observations noted in this study, participants in the yoga group appeared to report post-intervention improvements across multiple domains of the QOL-B questionnaire when compared with the control group. Areas such as respiratory symptoms, physical functioning, vitality, and role functioning showed favorable changes, which may suggest a potential association between yoga practice and enhanced quality of life. As a mind–body modality, yoga incorporates physical postures (asanas), breathing techniques (pranayama), and meditative or relaxation-based elements (e.g., dhyana), and may offer a complementary strategy to support well-being in this population. However, further studies are needed to clarify the consistency and clinical relevance of these preliminary findings (37–39). Yoga has been gradually explored as a potential non-pharmacologic approach that may support aspects of physical health, emotional balance, and psychological well-being in certain clinical contexts (37–40). Tan et al. (41) has been reported that yoga has a positive effect on dyspnea, sleep quality, and overall quality of life in patients with bronchiectasis, with improvements observed in dyspnea severity, sleep quality, and health-related quality of life scores over time compared to the control group. Through its holistic framework, yoga is thought to enhance parasympathetic activity, reduce sympathetic overactivation, and improve cardiopulmonary coordination, making it particularly suitable for individuals with chronic respiratory conditions (42–45). Over the past few decades, an increasing number of studies have investigated the potential role of yoga in managing respiratory conditions such as asthma (46, 47) and COPD (48, 49), with some reporting favorable outcomes. Some randomized controlled trials have suggested that yoga may be associated with improvements in select clinical outcomes among individuals with asthma (50) and COPD (51, 52). Reported benefits in these studies have included modest enhancements in pulmonary function parameters (e.g., FEV_1_, peak expiratory flow), reductions in symptoms such as dyspnea and wheezing, and improvements in psychological well-being and perceived quality of life. The potential benefits of yoga are thought to involve several physiological mechanisms, including enhanced thoracoabdominal movement, decreased airway resistance, improved respiratory muscle engagement, and possible modulation of neuroendocrine responses to stress. Among the components of yoga, breathing practices such as pranayama have been explored for their role in promoting ventilatory efficiency and mitigating dynamic hyperinflation, though the extent and consistency of these effects remain under investigation (53, 54). Although some encouraging results have been reported in other respiratory conditions, investigations specifically examining the role of yoga in individuals with bronchiectasis remain limited. Bronchiectasis represents a complex and heterogeneous condition, often involving symptoms such as chronic cough, sputum production, and recurrent respiratory infections, along with commonly reported fatigue and psychological burden (1, 8, 55–57). To date, relatively few exploratory or observational studies have investigated the potential role of yoga in individuals with non-cystic fibrosis bronchiectasis, and many have been limited by methodological constraints. The present study contributes to this emerging area by providing preliminary evidence that a structured yoga program may be associated with improvements in disease-specific quality of life and selected psychological measures. These findings should be interpreted cautiously and viewed as a basis for further investigation in more robustly designed trials. These findings underscore yoga’s potential not only to alleviate physical symptoms but also to address the psychological burden often overlooked in traditional disease management paradigms. Meanwhile, the observed improvements in the vitality and role functioning domains may reflect the capacity of yoga to mitigate fatigue and enhance physical endurance, which are often compromised in bronchiectasis due to chronic inflammation, impaired gas exchange, and poor exercise tolerance (55, 58, 59). Importantly, the deep relaxation and mindfulness elements integrated into the intervention may have helped reduce autonomic overactivation and chronic stress responses, which are increasingly recognized as contributors to fatigue and systemic malaise in chronic respiratory diseases (60).

Beyond its potential impact on health-related quality of life, yoga practice in this study appeared to be associated with favorable changes in certain psychological parameters. Our study showed that the intervention led to significant improvements in psychological outcomes, including reductions in anxiety and depression as measured by the HADS subscales. However, these improvements did not remain significant after correction for multiple comparisons. This lack of significance could be due to several factors, such as greater variability or measurement noise in these domains compared to other outcomes. Psychological outcomes like anxiety and depression are influenced by individual and contextual factors, including baseline psychological state, social support, and personal expectations, leading to more inconsistent effects. Furthermore, the correction for multiple comparisons, while necessary to control type I error, can reduce statistical power, particularly when effect sizes are smaller. As a result, the observed effects may not have been large enough to meet significance after adjustment. Future studies with larger samples, targeted interventions, and longer follow-up are needed to capture more consistent effects, particularly on anxiety and depression, and refine the understanding of the intervention’s impact on psychological health. These observations are broadly in line with prior research suggesting that yoga may contribute to improved psychological well-being. However, while several clinical studies have reported reductions in anxiety, depressive symptoms, and perceived stress following regular yoga practice in various populations, including individuals with chronic health conditions, the consistency and generalizability of such effects remain subjects of ongoing investigation (61–64). The potential psychological effects of yoga are thought to involve neurobiological and physiological pathways. Studies suggest that yoga may influence the HPA axis, lower cortisol levels, and increase vagal tone, promoting parasympathetic activity and improving emotional regulation. However, these mechanisms are not fully understood and likely vary based on individual and contextual factors (65–67). Additionally, yoga’s breath-centered and meditative aspects may enhance mindfulness, cognitive adaptability, and stress coping. Neuroimaging studies indicate that yoga could affect brain regions involved in emotional processing, such as the amygdala, hippocampus, and prefrontal cortex, though these effects are preliminary and require further exploration (68, 69). These neuroadaptive changes likely contribute to better affective control and reduced psychological distress. In bronchiectasis, where chronic symptoms and emotional vulnerability are prevalent, yoga may provide significant therapeutic benefits, complementing standard care and improving mental well-being. It is worth noting that participants in the yoga group reported higher post-intervention scores on the SF-36 physical component, which may suggest a perceived improvement in aspects of physical well-being. This finding further supports the hypothesis that yoga not only improves objective symptom burden but also fosters a more favorable perception of health and functioning, likely through a combination of physiological, behavioral, and emotional benefits.

It is important to consider the potential placebo or expectancy effects that may have influenced the outcomes observed in this study. Given that the intervention was delivered through online group sessions, participants’ expectations of improvement might have been influenced by their belief in the efficacy of yoga-like practices, rather than the intervention itself. These expectancy effects are common in studies involving behavioral interventions, particularly when participants are aware of the potential benefits. Furthermore, cultural factors may have played a role in shaping participants’ responses to the intervention. In the context of the Chinese patient population, the traditional acceptance of mind-body practices such as yoga or qigong may have influenced participants’ willingness to engage with the intervention and their perceptions of its effectiveness. The cultural background and prior experiences with similar practices could have contributed to heightened expectations and, potentially, improvements in psychological and health-related outcomes. While these factors do not diminish the value of the observed effects, they must be considered when interpreting the results. Future studies with more rigorous control groups and diverse patient populations could help isolate the true effects of yoga-like practices from placebo or expectancy influences. Additionally, exploring how cultural context shapes the response to such interventions could provide valuable insights into their broader applicability.

While the improvement in psychological outcomes is statistically significant, it appears clinically modest due to the small effect size. This is likely influenced by factors such as the intervention’s nature, the patient population, and the short follow-up period. Although the changes are statistically meaningful, their clinical relevance may be limited. Future studies with larger sample sizes and longer follow-up are needed to assess whether these modest improvements can lead to more substantial and sustained clinical impacts. We hope ongoing research will better clarify the clinical utility of such interventions in enhancing psychological well-being. Overall, while statistically significant, the modest size of the improvements suggests that the intervention may offer limited benefits in psychological outcomes within the study’s scope.

Despite its contributions, this study has several limitations that should be critically considered. First, the small sample size, particularly in the yoga group (n=24), limits the generalizability of the results, and the lack of a formal *A priori* power analysis may affect statistical power. Second, the online delivery of the intervention, while convenient, may have introduced variability in participant engagement, adherence, and practice consistency. Third, the short intervention duration and lack of long-term follow-up limit conclusions about the persistence and sustainability of the observed effects. Additionally, the absence of an active control group restricts the ability to control for expectancy bias. While outcome assessors were blinded, participant blinding was not feasible, which may have introduced some expectation-related bias. Furthermore, the results of this study may be influenced by the specific cultural and healthcare context in which it was conducted, and the applicability of these findings to other settings or populations may be limited. Lastly, the study outcomes were based solely on self-report, and the inclusion of objective physiological measures (e.g., lung function, biomarkers) would have strengthened the findings. Future research should involve larger, more diverse cohorts, longer follow-up, and include objective physiological measures (e.g., inflammation markers, heart rate variability) to better understand the mechanisms. Exploring yoga’s potential to complement pulmonary rehabilitation or self-management strategies also warrants further study.

In addition to the statistical significance of the observed changes, it is important to consider the minimal clinically important difference (MCID), which provides context for the practical relevance of these findings. The MCID represents the smallest change in a measurement that participants perceive as beneficial, and is particularly useful in determining whether observed improvements are meaningful from a clinical perspective (70, 71). While our study demonstrated significant improvements in psychological outcomes and health-related quality of life, we were unable to directly compare these changes against established MCID thresholds for the specific instruments used. For instance, the MCID for the SF-36 and QOL-B has been reported in previous studies. However, applying these benchmarks directly to our population of elderly SSNHL patients may not be fully appropriate due to differences in clinical characteristics and disease progression. Therefore, future studies should aim to compare the observed changes in psychological outcomes and quality of life against these MCID values to assess whether they meet clinically meaningful thresholds. By considering the MCID, we aim to provide a more comprehensive interpretation of our findings, addressing both statistical significance and clinical relevance. This approach will be important in future research to ensure that interventions not only produce statistically significant results but also lead to meaningful improvements in patients’ daily lives.

## 5 Conclusion

In summary, the findings of this study suggest that a structured 8-week yoga program may be associated with improvements in quality of life and selected psychological outcomes among individuals with non-cystic fibrosis bronchiectasis. However, these results should be considered preliminary, and further research is required to confirm the effectiveness of yoga in this population. Larger, multicenter, long-term randomized controlled trials are needed to establish its role as a complementary intervention in comprehensive, patient-centered bronchiectasis care.

## Data Availability

The raw data supporting the conclusions of this article will be made available by the authors, without undue reservation.

## References

[1] Chalmers JD Chang AB Chotirmall SH Dhar R Mcshane PJ. Bronchiectasis. Nat Rev Dis Primers. (2018) 1:45. doi: 10.1038/s41572-018-0042-3, PMID: 30442957

[2] Keir HR Chalmers JD. Pathophysiology of bronchiectasis. Semin Respir Crit Care Med. (2021) 4:499–512. doi: 10.1055/s-0041-1730891, PMID: 34261175

[3] De Angelis A Johnson ED Sutharsan S Aliberti S. Exacerbations of bronchiectasis. Eur Respir Rev. (2024) 33(173):240085. doi: 10.1183/16000617.0085-2024, PMID: 39048130 PMC11267293

[4] Nigro M Laska IF Traversi L Simonetta E Polverino E. Epidemiology of bronchiectasis. Eur Respir Rev. (2024) 33(174):240091. doi: 10.1183/16000617.0091-2024, PMID: 39384303 PMC11462313

[5] Mcdermott GC Sparks JA. Rheumatoid arthritis and bronchiectasis risk: additional evidence linking autoimmunity and airways disease. Chest. (2024) 6:1276–7. doi: 10.1016/j.chest.2024.02.016, PMID: 38852957

[6] Gao YH Zheng HZ Lu HW Li YY Feng Y Gu SY. Quality-of-life bronchiectasis respiratory symptom scale predicts the risk of exacerbations in adults with bronchiectasis: A prospective observational study. Ann Am Thorac Soc. (2024) 3:393–401. doi: 10.1513/AnnalsATS.202302-133OC, PMID: 37962906

[7] Terpstra LC Biesenbeek S Altenburg J Boersma WG. Aetiology and disease severity are among the determinants of quality of life in bronchiectasis. Clin Respir J. (2019) 8:521–9. doi: 10.1111/crj.13054, PMID: 31295770

[8] Umoh VA Alasia DD Akpan EE Jumbo HE Ekwere ME Umoh IO. Psychological distress and health-related quality of life among stable patients with bronchiectasis. Niger J Clin Pract. (2022) 2:144–52. doi: 10.4103/njcp.njcp_689_20, PMID: 35170439

[9] Keskin H Dirol H Ergin M. Anxiety and depression as possible criteria in the treatment of bronchiectasis. Turk Gogus Kalp Damar Cerrahisi Derg. (2021) 2:233–8. doi: 10.5606/tgkdc.dergisi.2021.20389, PMID: 34104517 PMC8167466

[10] Al Oweidat K Marie D Toubasi AA Jaber DZ Ahmed KE Abu Alragheb BO. The prevalence of anxiety and depression in bronchiectasis patients and their association with disease severity: a cross-sectional study. Sci Rep. (2023) 1:20886. doi: 10.1038/s41598-023-48276-1, PMID: 38017245 PMC10684858

[11] Nicola A Oancea C Barata PI Adelina M Mateescu T Manolescu D. Health-related quality of life and stress-related disorders in patients with bronchiectasis after pulmonary resection. J Pers Med. (2023) 13(9):1310. doi: 10.3390/jpm13091310, PMID: 37763078 PMC10532533

[12] Chang AB Fortescue R Grimwood K Alexopoulou E Bell L Boyd J. European Respiratory Society guidelines for the management of children and adolescents with bronchiectasis. Eur Respir J. (2021) 58(2):2002990. doi: 10.1183/13993003.02990-2020, PMID: 33542057

[13] Li Z Liu S Wang L Smith L. Mind-body exercise for anxiety and depression in COPD patients: A systematic review and meta-analysis. Int J Environ Res Public Health. (2019) 17(1):22. doi: 10.3390/ijerph17010022, PMID: 31861418 PMC6981896

[14] Cao A Feng F Zhang L Zhou X. Baduanjin exercise for chronic obstructive pulmonary disease: an updated systematic review and meta-analysis. Clin Rehabil. (2020) 8:1004–13. doi: 10.1177/0269215520926635, PMID: 32517512

[15] Ross A Thomas S. The health benefits of yoga and exercise: a review of comparison studies. J Altern Complement Med. (2010) 1:3–12. doi: 10.1089/acm.2009.0044, PMID: 20105062

[16] Govindaraj R Karmani S Varambally S Gangadhar BN. Yoga and physical exercise - a review and comparison. Int Rev Psychiatry. (2016) 3:242–53. doi: 10.3109/09540261.2016.1160878, PMID: 27044898

[17] Gautam S Kiran UV. Clinical effects of yoga and meditational practices on the holistic health of chronic kidney disease patients: A systematic review. Cureus. (2024) 4:e57546. doi: 10.7759/cureus.57546, PMID: 38707181 PMC11068214

[18] Woodyard C. Exploring the therapeutic effects of yoga and its ability to increase quality of life. Int J Yoga. (2011) 2:49–54. doi: 10.4103/0973-6131.85485, PMID: 22022122 PMC3193654

[19] Cramer H Lauche R Klose P Lange S Langhorst J Dobos GJ. Yoga for improving health-related quality of life, mental health and cancer-related symptoms in women diagnosed with breast cancer. Cochrane Database Syst Rev. (2017) 1:Cd010802. doi: 10.1002/14651858.CD010802.pub2, PMID: 28045199 PMC6465041

[20] Buran Cirak Y Yilmaz Yelvar GD Durustkan Elbasi N. Effectiveness of 12-week inspiratory muscle training with manual therapy in patients with COPD: A randomized controlled study. Clin Respir J. (2022) 4:317–28. doi: 10.1111/crj.13486, PMID: 35332685 PMC9060133

[21] Ranjita R Hankey A Nagendra HR Mohanty S. Yoga-based pulmonary rehabilitation for the management of dyspnea in coal miners with chronic obstructive pulmonary disease: A randomized controlled trial. J Ayurveda Integr Med. (2016) 3:158–66. doi: 10.1016/j.jaim.2015.12.001, PMID: 27545747 PMC5052394

[22] Dua R Malik S Bhadoria AS Neyaz O Krishnan AS Pandya C. Effectiveness of telemedicine interventions in chronic obstructive pulmonary disease (COPD) management: A randomized controlled trial comparing yoga therapy and pulmonary rehabilitation over three months. Cureus. (2024) 3:e56060. doi: 10.7759/cureus.56060, PMID: 38618447 PMC11009474

[23] Liu XC Pan L Hu Q Dong WP Yan JH Dong L. Effects of yoga training in patients with chronic obstructive pulmonary disease: a systematic review and meta-analysis. J Thorac Dis. (2014) 6:795–802. doi: 10.3978/j.issn.2072-1439.2014.06.05, PMID: 24977005 PMC4073384

[24] Donesky D Melendez M Nguyen HQ Carrieri-Kohlman V. A responder analysis of the effects of yoga for individuals with COPD: who benefits and how? Int J Yoga Therap. (2012) 22:23–36., PMID: 23070669

[25] Erdoğan Yüce G Taşcı S. Effect of pranayama breathing technique on asthma control, pulmonary function, and quality of life: A single-blind, randomized, controlled trial. Complement Ther Clin Pract. (2020) 38:101081. doi: 10.1016/j.ctcp.2019.101081, PMID: 32056817

[26] Streeter CC Whitfield TH Owen L Rein T Karri SK Yakhkind A. Effects of yoga versus walking on mood, anxiety, and brain GABA levels: a randomized controlled MRS study. J Altern Complement Med. (2010) 11:1145–52. doi: 10.1089/acm.2010.0007, PMID: 20722471 PMC3111147

[27] Kiecolt-Glaser JK Bennett JM Andridge R Peng J Shapiro CL Malarkey WB. Yoga's impact on inflammation, mood, and fatigue in breast cancer survivors: a randomized controlled trial. J Clin Oncol. (2014) 10:1040–9. doi: 10.1200/jco.2013.51.8860, PMID: 24470004 PMC3965259

[28] Cramer H Posadzki P Dobos G Langhorst J. Yoga for asthma: a systematic review and meta-analysis. Ann Allergy Asthma Immunol. (2014) 6:503–10.e5. doi: 10.1016/j.anai.2014.03.014, PMID: 24726198

[29] Joshi LN Joshi VD Gokhale LV. Effect of short term 'Pranayam' practice on breathing rate and ventilatory functions of lung. Indian J Physiol Pharmacol. (1992) 2:105–8., PMID: 1506070

[30] Vempati R Bijlani RL Deepak KK. The efficacy of a comprehensive lifestyle modification programme based on yoga in the management of bronchial asthma: a randomized controlled trial. BMC Pulm Med. (2009) 9:37. doi: 10.1186/1471-2466-9-37, PMID: 19643002 PMC2734746

[31] Martínez-García M De Gracia J Vendrell Relat M Girón RM Máiz Carro L de la Rosa Carrillo D. Multidimensional approach to non-cystic fibrosis bronchiectasis: the FACED score. Eur Respir J. (2014) 5:1357–67. doi: 10.1183/09031936.00026313, PMID: 24232697

[32] Chalmers JD Goeminne P Aliberti S Mcdonnell MJ Lonni S Davidson J. The bronchiectasis severity index. An international derivation and validation study. Am J Respir Crit Care Med. (2014) 5:576–85. doi: 10.1164/rccm.201309-1575OC, PMID: 24328736 PMC3977711

[33] Quittner AL O'donnell AE Salathe MA Lewis SA Li X Montgomery AB. Quality of Life Questionnaire-Bronchiectasis: final psychometric analyses and determination of minimal important difference scores. Thorax. (2015) 1:12–20. doi: 10.1136/thoraxjnl-2014-205918, PMID: 25323621

[34] Ware JE Jr. Sherbourne CD. The MOS 36-item short-form health survey (SF-36). I. Conceptual framework and item selection. Med Care. (1992) 6:473–83. doi: 10.1097/00005650-199206000-00002 1593914

[35] Zigmond AS Snaith RP. The hospital anxiety and depression scale. Acta Psychiatr Scand. (1983) 6:361–70. doi: 10.1111/j.1600-0447.1983.tb09716.x, PMID: 6880820

[36] Lee EH. Review of the psychometric evidence of the perceived stress scale. Asian Nurs Res (Korean Soc Nurs Sci). (2012) 4:121–7. doi: 10.1016/j.anr.2012.08.004, PMID: 25031113

[37] Gothe NP Khan I Hayes J Erlenbach E Damoiseaux JS. Yoga effects on brain health: A systematic review of the current literature. Brain Plast. (2019) 1:105–22. doi: 10.3233/bpl-190084, PMID: 31970064 PMC6971819

[38] Toutain M Marais AL Moscone AL Gauthier A Leconte P. Effects of yoga on mental health. Rev Prat. (2024) 9:1011–4., PMID: 39625032

[39] Chobe S Chobe M Metri K Patra SK Nagaratna R. Impact of Yoga on cognition and mental health among elderly: A systematic review. Complement Ther Med. (2020) 52:102421. doi: 10.1016/j.ctim.2020.102421, PMID: 32951703

[40] Ko KY Kwok ZCM Chan HY. Effects of yoga on physical and psychological health among community-dwelling older adults: A systematic review and meta-analysis. Int J Older People Nurs. (2023) 5:e12562. doi: 10.1111/opn.12562, PMID: 37577926

[41] Tan MS Algun ZC Duger M Aslan Keles Y. The effect of yoga on dyspnea, sleep, and quality of life in patients with bronchiectasis: A randomized controlled trial. Complement Ther Clin Pract. (2024) 57:101914. doi: 10.1016/j.ctcp.2024.101914, PMID: 39388786

[42] Nakayama N Kono A Moriwaki Y Niihara M Aizawa R Ookabe S. Improved sympathetic activity with short-term effects of yoga in young adults. Holist Nurs Pract. (2024). doi: 10.1097/hnp.0000000000000675, PMID: 39212538

[43] Vempati RP Telles S. Yoga-based guided relaxation reduces sympathetic activity judged from baseline levels. Psychol Rep. (2002) 2:487–94. doi: 10.2466/pr0.2002.90.2.487, PMID: 12061588

[44] Shobana R Maheshkumar K Venkateswaran ST Geetha MB Padmavathi R. Effect of long-term yoga training on autonomic function among the healthy adults. J Family Med Prim Care. (2022) 7:3471–5. doi: 10.4103/jfmpc.jfmpc_199_21, PMID: 36387716 PMC9648241

[45] Akhtar P Yardi S Akhtar M. Effects of yoga on functional capacity and well being. Int J Yoga. (2013) 1:76–9. doi: 10.4103/0973-6131.105952, PMID: 23439856 PMC3573548

[46] Das RR Sankar J Kabra SK. Role of breathing exercises in asthma-yoga and pranayama. Indian J Pediatr. (2022) 2:174–80. doi: 10.1007/s12098-021-03998-w, PMID: 34812995

[47] Yadav A Sindhwani G Kumari R Goel A Bisht K. Effect of adjuvant yoga therapy for asthma control: A randomized controlled trial. J Ayurveda Integr Med. (2024) 1:100847. doi: 10.1016/j.jaim.2023.100847, PMID: 38237454 PMC10828812

[48] Cai Y Ren X Wang J Ma B Chen O. Effects of breathing exercises in patients with chronic obstructive pulmonary disease: A network meta-analysis. Arch Phys Med Rehabil. (2024) 3:558–70. doi: 10.1016/j.apmr.2023.04.014, PMID: 37150427

[49] Deluca ND Vajta Gomez JP Vital I Cahalin LP Campos MA. The impact of yoga on inspiratory muscle performance in veterans with COPD: A pilot study. Int J Yoga Therap. (2021) 31(1):Article4. doi: 10.17761/2021-d-19-00066, PMID: 34044450

[50] Yadav A Kumari R Goel A Sindhwani G Singh N Khanduri R. Efficacy of a structured yoga intervention integrated with routine care versus exercise on pulmonary function and quality of life of asthma patients: A randomized controlled trial. Adv Mind Body Med. (2024) 2:10–5. doi: 10.1016/j.jaim.2023.100847, PMID: 38837777

[51] Kaminsky DA Guntupalli KK Lippmann J Burns SM Brock MA Skelly J. Effect of yoga breathing (Pranayama) on exercise tolerance in patients with chronic obstructive pulmonary disease: A randomized, controlled trial. J Altern Complement Med. (2017) 9:696–704. doi: 10.1089/acm.2017.0102, PMID: 28714735 PMC5610410

[52] Yudhawati R Rasjid Hs M. Effect of yoga on FEV1, 6-minute walk distance (6-MWD) and quality of life in patients with COPD group B. Adv Respir Med. (2019) 5:261–8. doi: 10.5603/arm.2019.0047, PMID: 31680225

[53] Nambi G Abdelbasset WK Elshehawy AA Eltrawy HH Abodonya AM Saleh AK. Yoga in Burn: Role of pranayama breathing exercise on pulmonary function, respiratory muscle activity and exercise tolerance in full-thickness circumferential burns of the chest. Burns. (2021) 1:206–14. doi: 10.1016/j.burns.2020.06.033, PMID: 32709430

[54] Mooventhan A Khode V. Effect of Bhramari pranayama and OM chanting on pulmonary function in healthy individuals: A prospective randomized control trial. Int J Yoga. (2014) 2:104–10. doi: 10.4103/0973-6131.133875, PMID: 25035619 PMC4097894

[55] Hester KL Macfarlane JG Tedd H Jary H Mcalinden P Rostron L. Fatigue in bronchiectasis. Qjm. (2012) 3:235–40. doi: 10.1093/qjmed/hcr184, PMID: 22016379

[56] Ozalp O Inal-Ince D Calik E Vardar-Yagli N Saglam M Savci S. Extrapulmonary features of bronchiectasis: muscle function, exercise capacity, fatigue, and health status. Multidiscip Respir Med. (2012) 1:3. doi: 10.1186/2049-6958-7-3, PMID: 22958327 PMC3415114

[57] Cox NS Wilson CJ Bennett KA Johnston K Potter A Chang AB. Health-related quality of life and psychological wellbeing are poor in children with bronchiectasis and their parents. ERJ Open Res. (2019) 5(3):00063-2019. doi: 10.1183/23120541.00063-2019, PMID: 31528635 PMC6734007

[58] José A Ramos TM De Castro RAS De Oliveira CS De Camargo AA Athanazio RA. Reduced physical activity with bronchiectasis. Respir Care. (2018) 12:1498–505. doi: 10.4187/respcare.05771, PMID: 30254043

[59] Cordova-Rivera L Gibson PG Gardiner PA Mcdonald VM. Physical activity associates with disease characteristics of severe asthma, bronchiectasis and COPD. Respirology. (2019) 4:352–60. doi: 10.1111/resp.13428, PMID: 30384396

[60] Tanaka M Tajima S Mizuno K Ishii A Konishi Y Miike T. Frontier studies on fatigue, autonomic nerve dysfunction, and sleep-rhythm disorder. J Physiol Sci. (2015) 6:483–98. doi: 10.1007/s12576-015-0399-y, PMID: 26420687 PMC4621713

[61] Kwok JYY Kwan JCY Auyeung M Mok VCT Lau CKY Choi KC. Effects of mindfulness yoga vs stretching and resistance training exercises on anxiety and depression for people with parkinson disease: A randomized clinical trial. JAMA Neurol. (2019) 7:755–63. doi: 10.1001/jamaneurol.2019.0534, PMID: 30958514 PMC6583059

[62] Liu W Liu J Ma L Chen J. Effect of mindfulness yoga on anxiety and depression in early breast cancer patients received adjuvant chemotherapy: a randomized clinical trial. J Cancer Res Clin Oncol. (2022) 9:2549–60. doi: 10.1007/s00432-022-04167-y, PMID: 35788727 PMC9253261

[63] Szaszkó B Schmid RR Pomper U Maiworm M Laiber S Tschenett H. The influence of hatha yoga on stress, anxiety, and suppression: A randomized controlled trial. Acta Psychol (Amst). (2023) 241:104075. doi: 10.1016/j.actpsy.2023.104075, PMID: 37931334

[64] Sharma N Sahni PS Sharma US Kumar J Garg R. Effect of yoga on the stress, anxiety, and depression of COVID-19-positive patients: A quasi-randomized controlled study. Int J Yoga Therap. (2022) 32(2022):Article8. doi: 10.17761/2022-d-22-00013, PMID: 35850136

[65] Kumar AP Dhamodhini KS Venugopal V Silambanan S Shah MK Admavathi RP. Role of yoga in stress management and implications in major depression disorder. J Ayurveda Integr Med. (2023) 5:100767. doi: 10.1016/j.jaim.2023.100767, PMID: 37741161 PMC10520539

[66] Pakulanon S Le Scanff C Filaire E Cottin F Rama L Teixeira A. Effects of yoga and mindfulness meditation on stress-related variables: A randomized controlled trial. Int J Yoga Therap. (2024) 34(2024):Article7. doi: 10.17761/2024-d-22-00021, PMID: 38952154

[67] Sarubin N Nothdurfter C Schüle C Lieb M Uhr M Born C. The influence of Hatha yoga as an add-on treatment in major depression on hypothalamic-pituitary-adrenal-axis activity: a randomized trial. J Psychiatr Res. (2014) 53:76–83. doi: 10.1016/j.jpsychires.2014.02.022, PMID: 24655586

[68] Villemure C Čeko M Cotton VA Bushnell MC. Neuroprotective effects of yoga practice: age-, experience-, and frequency-dependent plasticity. Front Hum Neurosci. (2015) 281:281. doi: 10.3389/fnhum.2015.00281, PMID: 26029093 PMC4428135

[69] Desai R Tailor A Bhatt T. Effects of yoga on brain waves and structural activation: A review. Complement Ther Clin Pract. (2015) 2:112–8. doi: 10.1016/j.ctcp.2015.02.002, PMID: 25824030

[70] Sedaghat AR. Understanding the minimal clinically important difference (MCID) of patient-reported outcome measures. Otolaryngol Head Neck Surg. (2019) 4:551–60. doi: 10.1177/0194599819852604, PMID: 31159641

[71] Molino J Harrington J Racine-Avila J Aaron R. Deconstructing the minimum clinically important difference (MCID). Orthop Res Rev. (2022) 53:35–42. doi: 10.2147/orr.S349268, PMID: 35210873 PMC8860454

